# Incidence of acute respiratory symptoms and COVID-19 infection in children in public schools in Bogotá, Colombia, from July to November, 2020

**DOI:** 10.7705/biomedica.6166

**Published:** 2022-10-31

**Authors:** José Moreno-Montoya, Diana Benavides-Arias, Luz Amparo Pérez, Jennifer Ruiz, Deidamia García, Iván Osejo, Edwin A. Ussa, Camilo Pino, Fernando Pío de La Hoz

**Affiliations:** 1 Subdirección de Estudios Clínicos, Fundación Santa Fe de Bogotá, Bogotá, D.C., Colombia Subdirección de Estudios Clínicos Fundación Santa Fe de Bogotá Bogotá, D.C. Colombia; 2 Departamento de Salud Pública, Universidad Nacional de Colombia, Bogotá, D.C., Colombia Universidad Nacional de Colombia Departamento de Salud Pública Universidad Nacional de Colombia Bogotá, D.C. Colombia; 3 Departamento de Psicología, Universidad Nacional de Colombia Bogotá, D.C., Colombia Universidad Nacional de Colombia Departamento de Psicología Universidad Nacional de Colombia Bogotá, D.C. Colombia; 4 Subsecretaría de Gestión Institucional, Secretaría de Educación Distrital, Bogotá, D.C., Colombia Subsecretaría de Gestión Institucional Secretaría de Educación Distrital Bogotá, D.C. Colombia; 5 Subsecretaría de Integración Interinstitucional, Secretaría de Educación Distrital, Bogotá, D.C., Colombia Subsecretaría de Integración Interinstitucional Secretaría de Educación Distrital Bogotá, D.C. Colombia; 6 Dirección de Bienestar Estudiantil, Secretaría de Educación Distrital, Bogotá, D.C., Colombia Dirección de Bienestar Estudiantil Secretaría de Educación Distrital Bogotá, D.C. Colombia; 7 Laboratorio de Zoología y Ecología Acuática LaZoea, Universidad de los Andes, Bogotá, D.C., Colombia Universidad de los Andes Laboratorio de Zoología y Ecología Acuática LaZoea Universidad de los Andes Bogotá, D.C. Colombia; 8 Grupo de Investigación en Artrópodos Kumangui, Universidad Distrital Francisco José de Caldas, Bogotá, D.C., Colombia Universidad Distrital Francisco José de Caldas Grupo de Investigación en Artrópodos Kumangui Universidad Distrital Francisco José de Caldas Bogotá, D.C. Colombia; 9 Dirección de Participación y Relaciones Interinstitucionales, Secretaría de Educación Distrital, Bogotá, D.C., Colombia Dirección de Participación y Relaciones Interinstitucionales Secretaría de Educación Distrital Bogotá, D.C. Colombia; 10 Laboratorio de Investigación en Sistemas Inteligentes, Facultad de Ingeniería, Universidad Nacional de Colombia, Bogotá, D.C., Colombia Universidad Nacional de Colombia Laboratorio de Investigación en Sistemas Inteligentes Facultad de Ingeniería Universidad Nacional de Colombia Bogotá, D.C. Colombia

**Keywords:** Coronavirus infections, respiratory tract infections, communicable diseases, epidemiology, surveillance, adolescent, child, infecciones por coronavirus, infecciones del sistema respiratorio, enfermedades transmisibles, epidemiología, vigilancia, adolescente, niño

## Abstract

**Introduction:**

More than 90% of children infected with COVID-19 worldwide developed mild to moderate disease. In Colombia, during 2020, COVID-19 infections in children stayed below 9.2% of the total cases, with no trends for age group or sex.

**Objective:**

To estimate the incidence of acute respiratory symptoms and COVID-19 in children from public schools in Bogotá, Colombia during the second semester of 2020.

**Material and methods:**

A telephone survey was conducted in over 5,000 scholar children. Antecedents and use of health services were informed. Descriptive statistics were used.

**Results:**

A total of 151.470 persons per day accounting for an IR of 157,8 per 100,000 people; almost three times the rate reported by the official surveillance system in the city.

**Conclusion:**

A lack of diagnosis and consultation in children was found compared to the general population. Further research is needed to elucidate the true burden of the disease in children.

## Resumen

**Introducción.:**

Más del 90% de los niños infectados con COVID-19 en el mundo, desarrollaron enfermedad leve a moderada. En Colombia, durante el 2020, la infección del COVID-19 en niños se mantuvo por debajo de 9,2 % del total de los casos sin tendencias por grupo de edad o sexo.

**Objetivo.:**

Estimar la incidencia de síntomas respiratorios agudos y COVID19 en niños de escuelas públicas en Bogotá (Colombia) durante el segundo semestre de 2020.

**Materiales y métodos.:**

Se hizo una encuesta telefónica en más de 5.000 escolares. Se recolectó información de antecedentes médicos y uso de servicios de salud. La información obtenida se describió mediante estadística descriptiva.

**Resultados.:**

Se contabilizó un total de 151.470 personas al día para una tasa de incidencia de 157,8 en 100.000 personas, casi tres veces la tasa reportada por el sistema de vigilancia oficial de la ciudad.

**Conclusión.:**

Se encontraron deficiencias en el diagnóstico y consulta de los niños, al compararlos con la población general. Se necesita más investigación para dilucidar la verdadera carga de la enfermedad en la población infantil.

## Introduction

It is estimated that children accounted for up to 15,7% of the total of COVID-19 infections around the world, with more than 90% of them developing mild to moderate symptoms ^1^. In Colombia, during 2020,

COVID-19 infections stayed below 9,2% of the total cases, with no trends by age group or sex. Mild, and asymptomatic cases contributed to 96,2% of the infections, with a death proportion of 0,16% ^2^.

Despite this, SARS-CoV-2 infection seems to be less frequent and severe in children ^3^. Actions designed to control its transmission have required radical changes in children’s normal lifestyles, including temporary shutdown of schools. However, it is partially unknown to what extent closing schools has protected children, since there is no specific data of its prevalence in this population, and national figures are based on general-population-based studies, especially in developing countries ^4^.

Here, we describe the incidence of COVID-19 infection in children enrolled in the public education system of Bogota, a large metropolitan area in Latin America ^5^, that closed schools in March, 2020**.**

## Materials and methods

Bogotá is the capital and major city of Colombia, with an estimated population above 7 million inhabitants, of which 31% are under 18 years of age ^6^.

Telephone surveys were conducted between August 11 and November 30, 2020, in a fully random sample without replacement of students enrolled in Bogotá’s public education system, which serves a population exceeding 800,000 students, and 62% of the city’s total scholar population. According to official figures, 10% attended preschool, 44% primary school, 30% secondary school, and 7% adult programs. Of all scholars, 51,52% were females. In the public school system in Bogotá, 87,8% of the students come from low or middle-low income groups ^7^. The population included in this study was not selected by any specific criteria. Therefore, any child ascribed to the public education system could be part of the sample.

An expected sample of n=4.784 students was estimated (maximum error=0,03, prevalence=0,5) using as sampling frame the total official list of children ascribed to public schools in the city in January 2020. It was expected that more than 99% of the students or caregivers in Bogotá have an active phone line at the time of this study ^5^. The survey was part of routine promotion activities conducted by *Secretaría de Educación de Bogotá*, which includes activities for health promotion and disease prevention, academic remote assistance for students, and virtual meetings with parents and caregivers ^5^. Therefore, ethical committee approval was not required, though verbal consent was obtained from caretakers.

We obtained information about the clinical history of respiratory symptoms, use of health services, COVID-19 diagnosis in the last month, age, gender, and history of known household contacts with COVID-19 cases or deaths. No active surveillance was carried out, so children were only interviewed as ordered by their insurer. All the information was consolidated in an anonymized database. For children under 12 years old, responses were given by the caregiver or parent registered in the database. Children over 12 years old, answered the interview personally but were assisted by their parents in all cases.

Incidence rates (IR) of COVID-19-like illness and confirmed cases per child per day were estimated during the period between July 12 to November 30, 2020. To obtain the IR denominator, we assumed that every participant in the sample contributed during 30 days of observation and, subsequently, that number was added for each student interviewed, yielding 151.470 person days of follow-up. Thus, every person/caregiver was asked to provide information regarding respiratory symptoms or COVID-19 confirmed infection during the last 30 days prior to the interview. Also, Bogota’s IR for COVID-19 confirmed cases was estimated using data from the city’s surveillance system. There were 1,486,719 people from 3 to 17 years old living in Bogota during the 111 days of the study period, yielding 165,025,809 persons days of follow-up. Confidence intervals (CI_95%_) were estimated using a Poisson approximation to the normal distribution.

## Results

A total of 5.049 students were included in the sample (the excluding rate was lower than 20%, which included wrong registered numbers. No other additional information was provided regarding exclusion criteria). The ages ranged from 3 to 17 years old (average=10,56 years; CI_95%_ 10,51-10,61), being around 50,58% males.

Acute respiratory symptoms presented during the last month were reported by 239 participants, resulting in an IR of 157,8 cases per 105 person-days (CI_95%_ 138,4-179,1). Forty-five (18,81%) individuals attended health services, and 18,82% of the children who seemed to be ill, searched for medical assistance (Table 1). In total, 239 children underwent molecular assays for COVID-19 infection detection. Reasons for being tested included: having a confirmed case of a household contact (n=118), being symptomatic (n=24), and for unknown reasons (n=97). Sixty out of 239 (25,1%, CI_95%_ 19,74- 31,52%) resulted positive which yielded an IR of 39,61 cases per 105 person- days (CI_95%_30,23-50,99 per 105 person-days). Testing was performed by the surveillance system of the *Secretaría de Salud de Bogotá.*

**Table 1:** Symptoms and Covid-19 incidence

**Symptoms** ^a^	Under 14 years old N=3674 (n) %	14+ years old N= 1375 (n) %	**Total (P value)**
Last month respiratory symptoms	182 (4.95)	57 (4.14)	4.73% (0.25)
In symptomatic children: Last month medical consultation from respiratory symptoms /people who attended medical consultation last month.	34/182 (18,68)	11/57 (19,29)	18.82% (0.93)
		
		
		
In symptomatic children: COVID-19 tests	15/182 (8.24%)	9/57 (15.79%)	10.04% (0.14)
Children with symptoms last month	n=182	n=57	n=239
Cough	14	1	
Fever	4	1	
Nasal congestion	19	7	
Respiratory distress	17	7	
Chest pain	1	2	
General discomfort	4	2	
Excessive tiredness	1	2	
Has had any contact with a COVID-19	229/3674 (6.23%)	92/1375 (6.69%)	6.35% (0.57)
patient?			
If yes, the child was tested?	78/229 (34.06%)	40/92 (43.47%)	36.76% (0.28)
Does the child live with some COVID-19	176/3674 (4.79%)	63/1375 (4.65%)	5.75% (0.84)
patient?			
If yes, the child was tested?	72/176 (40.9%)	30/63 (46.87%)	42.67% (0.56)
Somebody in your family has died from COVID-19?	114/3674 (3.1%)	33/1375 (2.4%)	2.91% (0.19)
COVID-19 Tests (Total)	151/3674 (4.1%)	88/1375 (6.4%)	4.73% (0.01)
Positives	37/151 (24.5%)	23/88 (26.13%)	25.1% (0.82)

^a^Not all the respondents answered the symptoms questions and those who answered could choose one or more options.

Table 2 shows acute respiratory disease and COVID-19 monthly incidents in children. No severe infections, hospitalization, or deaths were reported. In Bogota, the surveillance system detected 24,878 cases for the same age group during the study period (IR: 15,1 cases per 105 person-days, CI_95%_ 14,89-15,26 per 105 person-days) which was 61,87% lower than the estimated value from the survey.

**Table 2:** Monthly incidence of ARS and COVID-19 among children during the period July and November 2020 per 105 persons day.

Month	Cases ARS	Cases COVID-19	Persons at risk	IR- ARS*	IR- COVID-19**	n
July	35	1	17580	199.09	5.69	586
August	75	8	51810	144.76	15.44	1727
September	145	24	79920	181.43	30.03	2664
October	196	34	113940	172.02	29.84	3798
November	239	60	151470	157.79	39.61	5049

^*^Incidence rate of Acute Respiratory Symptoms

^**^Incidence rate COVID-19

## Discussion

This is one of the few studies, if any, reporting the incidence of COVID-19 in children using a population-based survey. The main finding is that children and adolescents staying home because of school closures are not at zero risk of acquiring COVID-19 infection. The incidence was 2,62 times bigger than the value reported by the city’s surveillance system for general population in the same group age and period.

These results raise the question of the effectiveness of school closure to protect children from COVID-19 infection and highlights the need for surveillance studies regarding other related problems derived from this measure such as mental health, decrease in physical activity, dietary changes, and social performance difficulties ^8^.

On the other hand, it showed a potential important underestimation of COVID-19 infection among children and adolescents in Colombia, given the low number of people who were tested even being symptomatic (24/239: 10,04%). Likewise, only 42.67% of children living with COVID-19 positive adults underwent molecular detection, adding-up to the underestimation proportion.

Besides this, unexplored barriers to access to health services and molecular testing contributes substantially to underrating ^5^. These findings could be associated with the fact that during pandemics, the screening efforts were concentrated on elderly, and morbid people. In addition, underrepresented minorities, immigrants, and low-income families could have been disproportionally affected by COVID-19 pandemics, worsening living and health conditions of school-age children ^9^.

No severe disease was reported in our survey, which is consistent with low risk of complications reported for this age group by global literature. However, despite that in children the infection is milder, the prognosis is better, and the mortality rate is lower compared to adult patients, children are potential carriers that could easily transmit the infection among the entire population. Regarding the above, in age groups below and above 14 years old, the positivity proportion of COVID-19 tests were similar, alike as the number of children living with somebody diagnosed for COVID-19.

However, the proportion of tests performed for each group was different, revealing different levels of prioritization during the surveillance, when the role of the children regarding transmission was not completely clarified. This reinforces the importance of early identification and intervention of COVID-19 in pediatric patients to control the pandemic, with further screening for other symptoms, such as gastrointestinal or psychological ones, which are common among children.

Further research is needed to elucidate the true burden of disease among children and the extent of the damage associated with school closures and lockdown measures, aimed to control the spread of the SARS-Cov-2 virus. New public health strategies for diagnosing and reducing its transmission in children and adolescents are required in order to diminish the potential risk of infection underestimated in this population, as well as to prevent collateral damages in other populations at risk. In addition to the demands of homeschooling, families in Bogotá need to be taught to meet the additional, and often complex educational and medical requirements of their children regarding the new normality in pandemic times.
